# Ultraviolet radiation causes leaf warming due to partial stomatal closure

**DOI:** 10.1093/hr/uhab066

**Published:** 2022-01-19

**Authors:** Tom B Williams, Ian C Dodd, Wagdy Y Sobeih, Nigel D Paul

**Affiliations:** 1 Lancaster Environment Centre, Lancaster University, Lancaster LA1 4YQ, UK; 2 Arid Agritec Ltd. Gordon Manley Building, Lancaster Environmental Centre, Lancaster University, Lancaster LA1 4YQ, UK

## Abstract

Variation in solar ultraviolet radiation induces a wide-range of plant responses from the cellular to whole-plant scale. We demonstrate here for the first time that partial stomatal closure caused by ultraviolet radiation exposure results in significant increases in leaf temperature. Significant leaf warming in response to ultraviolet radiation was consistent in tomato (*Solanum lycopersicum* L.) across different experimental approaches. In field experiments where solar ultraviolet radiation was attenuated using filters, exposure to ultraviolet radiation significantly decreased stomatal conductance and increased leaf temperature by up to 1.5°C. Using fluorescent lamps to provide ultraviolet radiation treatments, smaller but significant increases in leaf temperature due to decreases in stomatal conductance occurred in both multi-day controlled environment growth room experiments and short-term (<2 hours) climate cabinet irradiance response experiments. We show that leaf warming due to partial stomatal closure is independent of any direct warming effects of ultraviolet radiation manipulations. We discuss the implications of ultraviolet radiation-induced warming both for horticultural crop production and understanding broader plant responses to ultraviolet radiation.

## Introduction

The last three decades have transformed our understanding of many plant responses to ultraviolet (UV) radiation (280-400 nm) [1]. The initial focus on UVB (280-320 nm), driven by concern over increases in UVB due to stratospheric ozone depletion [2, 3], has evolved to recognize that variation in ambient UV, including UVA (320-400 nm) as well as UVB [4], affects many aspects of plant biochemistry, physiology and morphology [5], including crop growth, yield and quality [5, 6]. Horticultural crop production, especially of fresh fruit, vegetables and salads, has exploited these plant responses to UV by using novel cladding plastics with contrasting UV transmission properties [7, 8] or UV emitting LEDs [9] to regulate growth and improve crop coloration, taste, or pest and disease resistance. While horticultural claddings such as glass and “traditional” polymers such as polyethylene are typically opaque to UVB and shorter wavelength UVA radiation [7], cladding materials that transmit the full range of solar UV radiation, referred to here as UV transparent (UV-T), are becoming widely used by commercial growers.

One widely investigated response to UV radiation is reduced stomatal conductance (g_s_: Table 1). The magnitude, and even the direction of stomatal responses to UV, vary between species, the nature of UV treatments and other environmental conditions [10]. However, in most cases increases in UVB within and above the ambient range lead to reduced g_s_ through stomatal closure or, in some cases, decreased stomatal density (Table 1). Significant reductions in stomatal conductance in response to UV have been observed over timescales as short as a few hours but persist over weeks, months or, in a few studies, when UV treatments have been maintained over multiple growing seasons (Table 1). As with many UVB responses, stomatal closure is regulated by the UVB-specific photoreceptor uvr8 via mechanisms that also involve the generation of nitric oxide and hydrogen peroxide [11].

**Table 1:** Overview of published reports where exposure to UV radiation significantly reduced stomatal conductance or stomatal aperture. As noted in the main text, there are some reports of increased conductance or aperture in response to UV radiation, but this table illustrates the diversity of species (from woody plants (including conifers) to both dicotyledonous and monocotyledonous herbaceous species), experimental conditions, and timescales in which stomatal closure in response to UV has been observed

Species	Experimental method	Time-scale	Response to exposure to UV radiation	Reference
*Vaccinium corymbosum*	Filtered sunlight in the field (38°S)	Months	Stomatal conductance increased by exclusion of solar UVB	22
*Fagus sylvatica*	Filtered sunlight and lamps in the field (49°N)	Months	Stomatal conductance increased by the exclusion of solar UVB and decreased by UV supplementation	23
*Colocasia esculenta*	Filtered sunlight in the field (6/7°N)	Months	Stomatal conductance increased by exclusion of solar UVB	24
*Gossypium hirsutum Triticum aestivum Amaranthus tricolor Sorghum bicolor*	Filtered sunlight in the field (22°N)	Months	Stomatal conductance increased by exclusion of solar UVB. in all four species	25
*Vitis vinifera*	Filtered sunlight in the field (42°N)	Months	Stomatal conductance increased by exclusion of solar UVB	26
*Glycine max*	Lamps in the field (25°N)	Months	Stomatal conductance decreased by UV supplementation	27
*Abies faxoniana Picea asperata Swida hemsleyi Acer mono*	Lamps in the field (31°N)	Months	Stomatal conductance decreased by UV supplementation in all except *Abies faxoniana*	28
*Zea mays*	Lamps in the field (41°N)	5 weeks	Stomatal conductance decreased by UV supplementation.	29
*G. max*	Lamps in the glasshouse	4 weeks	Stomatal conductance decreased by exposure to UV radiation in 3 of 4 cultivars	13
*Pisum sativum Commelina communis Brassica napus*	Lamps in the glasshouse	5–30 days	Stomatal conductance decreased by exposure to UV radiation	30
*Oryza sativa*	Lamps in controlled environment	7 days	Stomatal conductance decreased by exposure to UV radiation	12
*Chenopodium quinoa*	Lamps in controlled environment	3 days	Stomatal conductance decreased by exposure to UV radiation	31
*Arabidopsis thaliana*	Lamps in controlled environment and in vitro	3–12 hours	Stomatal conductance and aperture reduced progressively with increasing UVB fluence rate	11
*Vicia faba*	Lamps in vitro	4 hours	Stomatal aperture reduced progressively with increasing duration of UVB exposure	32

The consequences of reduced g_s_ due to UVB has been widely discussed in relation to the wider effects of UVB on photosynthesis, transpiration and water-use-efficiency [12–14]. Variation in g_s_, by regulating transpirational cooling, also has a substantial and well-defined role in controlling leaf temperature [15, 16]. Increased leaf temperature has been associated with reduced g_s_ in response to environmental factors including drought [17], elevated CO_2_ concentration [18] and herbivory [19, 20]. Despite many reports of UV-induced stomatal closure across species and time-scales (Table 1), it is surprising that we are unaware of any indicating that UV-induced stomatal closure significantly increases leaf temperature. However, over recent years, we have received repeated anecdotal reports from commercial growers that crops cultivated under UV-T cladding grew more rapidly and matured earlier than crops grown under “traditional” plastics. Growers associated this earlier maturity with increased leaf temperature under UV-T films. We confirmed this observation at a commercial tomato farm in Antalya, Turkey. Leaf temperature of tomato plants cultivated under UV-T films since transplanting was substantially higher (1.9 ± 1.3**°**C**:** p < 0.05) than under standard cladding [21].

Here we tested the hypothesis that UV-induced partial stomatal closure decreases transpiration rates thereby increasing leaf temperature. We investigated this using different measurement approaches and at a range of scales in three experimental campaigns in contrasting environmental conditions. The UV component of sunlight was attenuated using plastic claddings in polytunnels in four repeat, multi-day experiments. In three repeat controlled environment (CE) growth room experiments of similar experimental duration, fluorescent tubes provided the UV treatments. Furthermore, 15 experiments using a range of irradiances and UV sources investigated short-term (<2 hours) responses in CE climate cabinets. Under this wide range of conditions, UV radiation caused partial stomatal closure thereby increasing leaf temperature. Differences in total incident radiation determined the magnitude of leaf warming, with greater effects in the field, up to approximately 1.5**°**C in our experiments, than under controlled environment conditions. While our experiments are relatively short compared with typical cropping cycles, the mechanistic links between UV-induced reductions in stomatal conductance, the established literature of medium-long term stomatal conductance responses to UV, and the links between gas exchange and leaf temperature, have broad implications within horticultural crop production.

## Results

### Solar UV radiation increases leaf temperature and decreases stomatal conductance in field experiments

Leaf temperature was significantly greater in UV+ (with UV radiation) polytunnels in all four experiments (Table S1), with no significant (p = 0.13) experiment x treatment interaction (Table S2a). UV treatment and experiment main effects were significant (p < 0.01) for both leaf temperature and g_s_ (Table S2a). Although g_s_ showed a significant main effect, there was a significant experiment x treatment interaction for g_s_ (p = 0.001; Table S2a) associated with contrasting weather conditions, which varied both between and in some cases within experiment. For example, across the four experiments total daily solar radiation dose (400-800 nm) varied between approximately 2000 and 7000 W m^−2^ d^−1^ and leaf temperature between 16 and 36°C. Here, detailed data are reported for the experiment completed when conditions were most consistently cloud-free (25 June – 3 July 2018).

**Figure 1:**
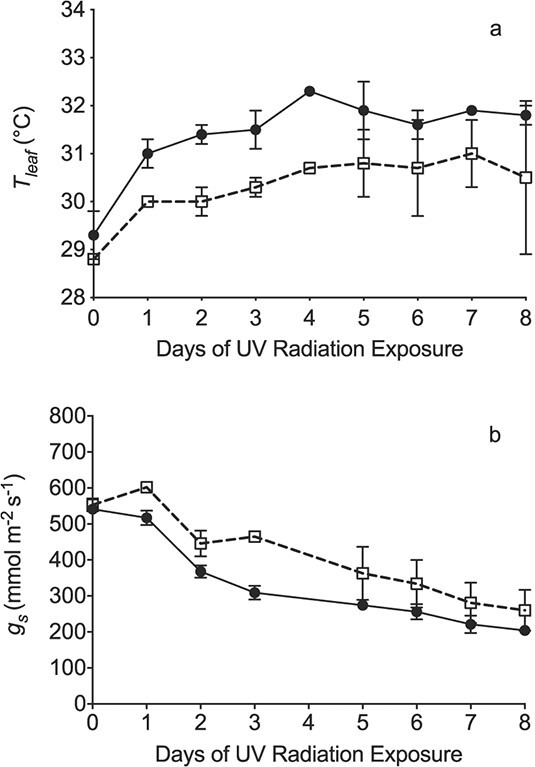
Time courses of (a) leaf temperature (T_leaf_), and (b) stomatal conductance (g_s_) under UV+ (closed circles) and UV- (open squares) treatments, for the field experiment completed when conditions were most consistently cloud-free (25 June – 3 July 2018). Each symbol is the mean of 20 replicate leaves (n = 20). Error bars represent ±1 SE. See Table S2b for full statistical analysis.

**Figure 2:**
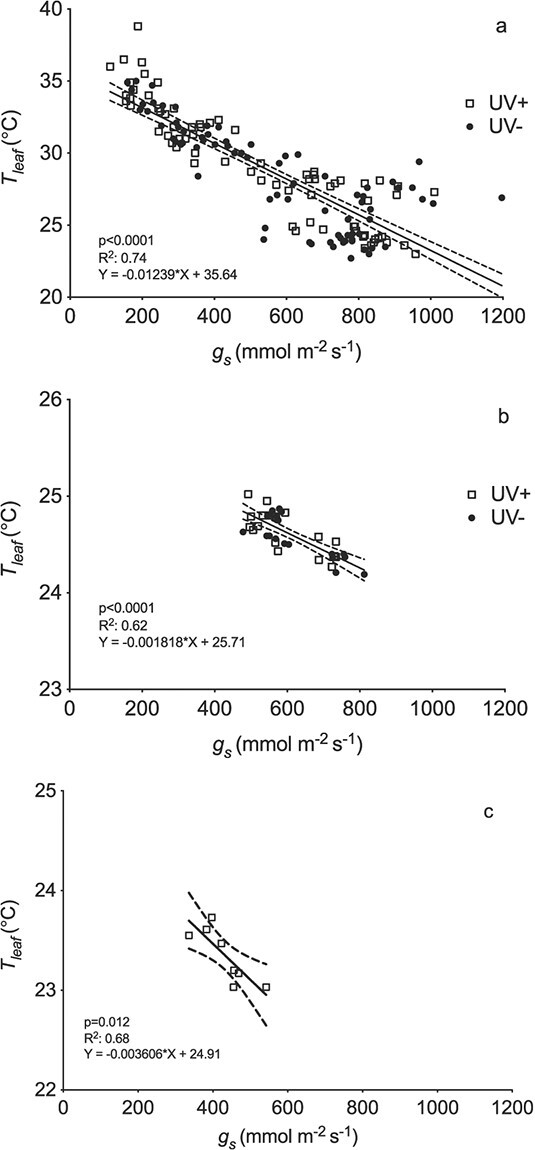
Linear regression analysis of the relationship between mean stomatal conductance (g_s_) and mean absolute leaf temperature (T_leaf_) in, (a) all four polytunnel experiments (n = 76), (b) all three controlled environment growth room experiments (n = 18), and (c) the climate cabinet experiment (n = 8). The results of linear regression analysis are summarised. For the polytunnel and growth room experiments data for both UV+ and UV- are presented. The slopes and intercepts of the two regressions were compared statistically and since there were no significant differences in either slopes (p = 0.07 and p = 0.99 for polytunnel and growth room experiments respectively) or intercepts (p = 0.27 and p = 0.77 respectively) pooled lines are shown. For the climate cabinet experiment, data for the irradiance closest to that in the CE growth room experiment are presented to facilitate comparison. In all cases the 95% confidence intervals are also shown. Each symbol is the mean of a separate individual leaf.

UV treatment had significant (p < 0.01; Table S2b) effects on both leaf temperature (T_leaf_) and g_s_. Day significantly affected g_s_ (p < 0.001) but not T_leaf_ (p = 0.13), with no significant (p > 0.05) polytunnel effects for either variable (Table S2b). There were no significant interactions (all p > 0.05), except for a treatment x polytunnel interaction for T_leaf_ only (p = 0.017; Table S2b). This positional effect occurred only in this experiment, and was not significant in the overall analysis. Despite day-to-day variation in leaf temperature and g_s_, the overall effects of UV treatment were robust, with a mean leaf temperature increase of 1.1 ± 0.9°C across all eight days, reaching 1.6 ± 0.7°C on Day 4 (Figure 1a). The UV+ treatment decreased g_s_ by a maximum of 156 ± 88 mmol m^−2^ s^−1^ (mean ± SE) on Day 3 (data not available for Day 4), with the mean decrease across the whole experiment at 86 ± 65 mmol m^−2^ s^−1^ (Figure 1b). There were highly significant positive linear relationships between T_leaf_ and g_s_ in both treatments (p < 0.001 for both UV+ and UV-: Figure 2a). Further analysis showed no significant differences between UV+ and UV- in either the slope or intercept of these relationships, allowing a single regression to the fitted to data from both treatments (p < 0.001: Figure 2a).

Mean air temperature between UV+ and UV- tunnels did not significantly differ (0.21°C: p = 0.53) during experimental measurements. Moreover, there was no significant relationship between differences in leaf temperature and differences in air temperature (p = 0.59). Thus, leaf temperatures were significantly higher under UV+ than UV- cladding, and this was associated with decreased g_s_ but not increased air temperature.

### UV radiation increases leaf temperature and decreases stomatal conductance in controlled environments

Analysis of data combined across all three growth room experiments (Table S3) showed that the main effect of experiment was significant for each leaf physiological parameter (p < 0.001; Table S4). Since there was no significant treatment x experiment interactions (p > 0.05; Table S4), the pooled data across all three experiments were used for further analysis.

**Figure 3:**
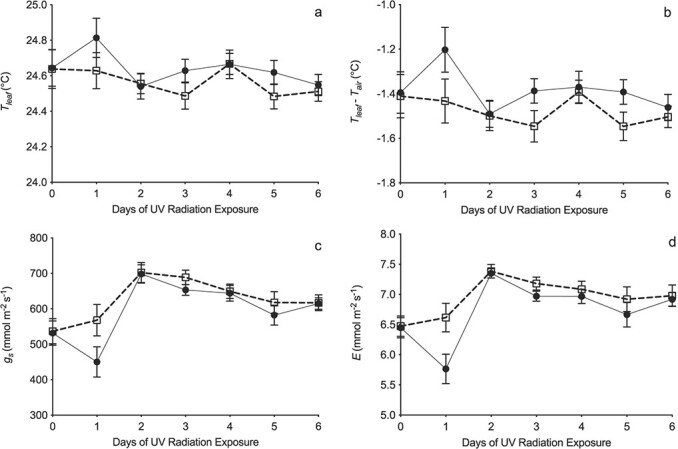
The response to UV+ (closed circles and solid line) and UV- (open squares and dashed line) treatments of (a) leaf temperature (T_leaf_), (b) relative leaf temperature (T_leaf_-T_air_), (c) stomatal conductance (*g_s_*), and (d) transpiration rate (*E*), when all three controlled environment growth room experiments were combined and analysed together. Cuvette temperature was held at 25**°**C. Each symbol is the mean of 18 replicate leaves: error bars represent ±1 SE but if not visible they were smaller than the symbol. See Table S4 for full statistical analysis.

Leaf temperature was higher with (UV+) than without (UV-) UV radiation, but this difference was not quite significant (p = 0.08; Table S4; Figure 3a). Leaf temperature measured relative to air temperature (T_leaf_- T_air_) was significantly (p = 0.007) higher in UV+ than UV- (Figure 3b), and varied between days (p = 0.003), although treatment differences were not affected by this (treatment x day: p = 0.14; Table S4). The largest difference in T_leaf_- T_air_ measured between treatments was 0.23 ± 0.20°C (Day 1: Figure 3b). The pattern of day-to-day variation in T_leaf_- T_air_ was not consistent across each experiment (experiment x day: p < 0.001; Table S4), likely due to small differences in transpiration rate. There was also no interaction between day, experiment and treatment (p = 0.43; Table S4). Stomatal conductance (Table S4; Figure 3c) and transpiration rate (Table S4; Figure 3d) were both significantly reduced in the presence of UV radiation (p = 0.008 and p = 0.002 respectively). Transpiration and g_s_ responses to UV treatment varied significantly between days (p < 0.001 for each; Table S4), with g_s_ up to 118 ± 47 mmol m^−2^ s^−1^ lower and transpiration rate up to 0.85 ± 0.49 mmol m^−2^ s^−1^ lower under UV+ than UV- (Figure 3c, d).

**Figure 4:**
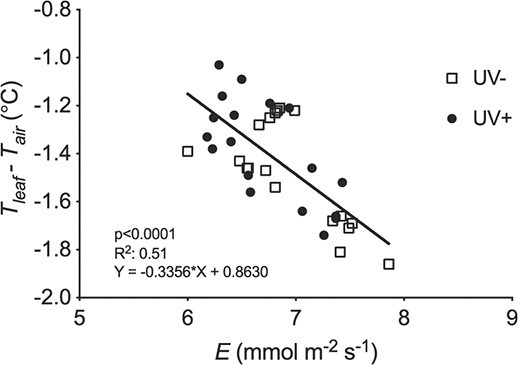
Linear regression analysis of the mean transpiration rate (*E*) and leaf temperature (*T_leaf_ - T_air_)* response to UV+ (closed circles) and UV- (open squares) treatments for the three controlled environment growth room experiments combined. Each symbol represents a separate individual leaf (n = 18). Linear regressions were initially fitted to the two datasets but the slopes and Y intercepts of the treatments were not significantly different (p = 0.92 and p = 0.78 respectively). As a result, the single linear regression fitted to the pooled data is presented (solid line).

There were highly significant linear relationships between T_leaf_- T_air_ and g_s_ for both UV+ and UV- (both p < 0.001). Further analysis showed no significant between-treatment differences in either the slopes (p = 0.99) or intercepts (p = 0.77), allowing a single highly significant pooled relationship (r^2^ = 0.62, p < 0.001) to be fitted to all data (Figure 2b). Similarly, there were no significant treatment differences in the slopes (p = 0.92) or intercepts (p = 0.78) of the linear regressions between T_leaf_- T_air_ and transpiration rate. The highly significant pooled relationship (r^2^ = 0.51, p < 0.001) fitted to all data (Figure 4) indicated that T_leaf_- T_air_ changed per unit transpiration by −0.34 ± 0.06°C per mmol m^−2^ s^−1^. Thus, as in the field, UV exposure significantly increased leaf temperature in the growth room, which was highly significantly linearly related to decreased g_s_, and also to decreased transpiration.

#### Increased leaf temperature and decreased stomatal conductance depend on UV irradiance.

As described in Materials and Methods, irradiance response data for g_s_ and transpiration rate (Table S5) are presented as the percentage change over the course of UV radiation application for each parameter.

**Figure 5:**
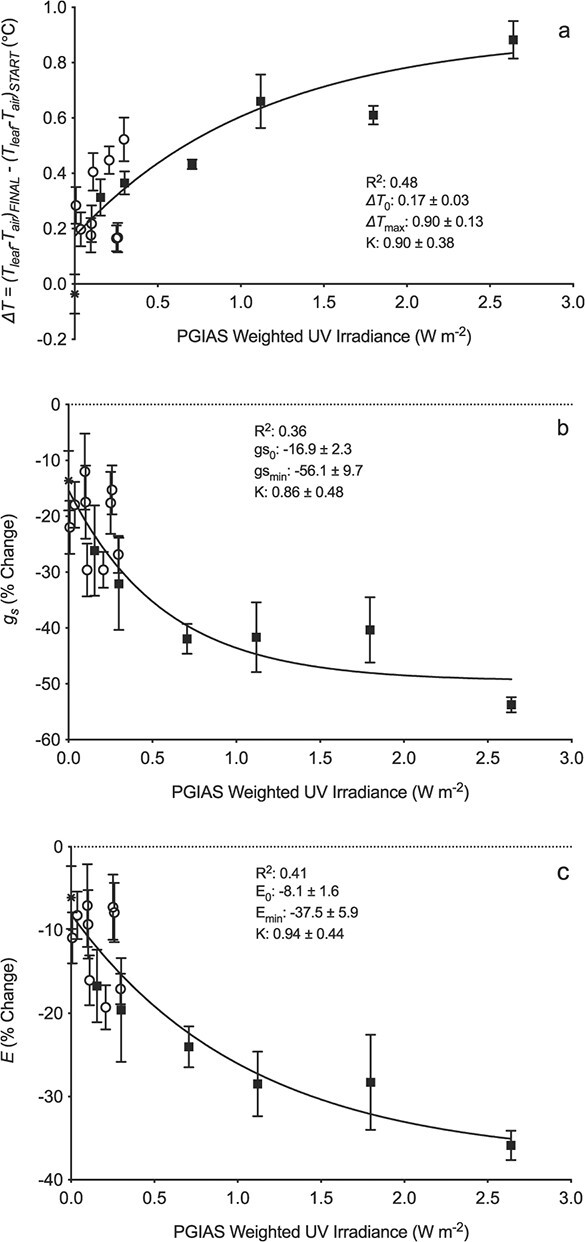
The climate cabinet UV irradiance response of (a) relative leaf temperature (*ΔT = (T_leaf_-T_air_)_FINAL_ - (T_leaf_-T_air_)_START_*), (b) stomatal conductance (*g_s_*), and (c) transpiration rate (*E*). The data are fitted with non-linear regression models (solid line) such that the response (Y) changes with increasing irradiance from the response at zero irradiance (Y_0_: the intercept on the Y axis) to reach a plateau (Y_max_ or Y_min_ respectively where Y increases or decreases with increasing irradiance) as a function of an irradiance response constant (K: a measure of the unit response per unit irradiance). All UV irradiances are weighted by the plant growth inhibition action spectrum (UV_F&C_; Flint & Caldwell, 2003). The symbols represent control (asterisk), filtered (open) and unfiltered (closed) UV treatments. The statistics of the regression lines are summarised on each individual plot. Error bars represent ±1 SE but if not visible they were smaller than the symbol. Each symbol is the mean of either 4 or 8 leaves, depending on the treatment (n = 4 or 8).

Leaf temperature difference (ΔT) increased significantly as a non-linear function of UV_F&C_ irradiance (Table S5; Figure 5a). In these controlled climate cabinet conditions, the plateau of the irradiance response predicted that UV treatments should increase ΔT by a maximum of 0.90 ± 0.13°C (Figure 5a). In comparison, the highest experimental UV_F&C_ irradiance increased ΔT by 0.88 ± 0.07°C (Figure 5a), while leaf excision increased ΔT by a maximum of 1.14°C. Stomatal conductance (Figure 5b) and transpiration rate (Figure 5c) both decreased significantly as a non-linear function of UV_F&C_ irradiance. In the climate cabinet, the non-linear model predicted that UV radiation should decrease g_s_ by a maximum of 56.1 ± 9.7% compared to the largest observed reduction of 53.8 ± 1.4% (Figure 5b). The predicted maximum reduction in transpiration rate due to UV radiation was 37.5 ± 5.9%, compared with largest observed reduction in response to UV treatments of 35.9 ± 1.8% (Figure 5c). Thus, observed reductions in g_s_, transpiration rate and ΔT are very similar to those predicted by the non-linear models.

#### Separating the effects of UV-induced stomatal closure on leaf temperature from other factors.

**Figure 6:**
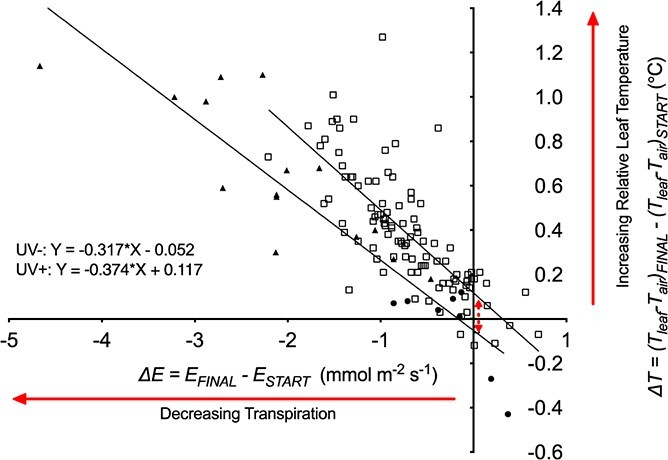
The change in relative leaf temperature (*ΔT = (T_leaf_-T_air_)_FINAL_ - (T_leaf_-T_air_)_START_*), plotted against the change in transpiration rate (*ΔE = E_FINAL_ - E_START_*) in response to the 15 UV treatments and one leaf excision experiment. Each symbol represents a separate individual leaf. The “UV-” data are derived from all non-irradiated treatments, including un-irradiated (closed circles) plus the excised leaves (closed triangles) that were undertaken to demonstrate the maximum ΔT increase possible in the controlled experimental environment (climate cabinet). These UV- data were plotted separately to data from all UV treated leaves (UV+: open squares). Linear regressions were fitted separately to the UV- and UV+ data. The two fitted regression were highly significant (both p < 0.001). The slopes of the fitted lines for the two datasets (a measure of warming due to reduced transpiration caused by partial stomatal closure) were not significantly different (p = 0.09) but the intercepts on the ΔT axis were highly significantly different (p < 0.001). This difference in intercept is the vertical offset between the linear regression lines when there is no difference in transpiration rate (indicated by the double-headed arrow). We interpret this as direct radiative heating from the UV lamps.

Enclosing the leaf inside the cuvette led to small changes in T_air_ and small decreases in g_s_ and transpiration rate even in the absence of UV treatments. Changes in T_air_ over the 90 minutes of measurements were an order of magnitude smaller than changes in T_leaf_ (mean changes in T_air_ were 0.04°C and 0.02°C for UV- and UV+ respectively compared with 0.38 and 0.41 for T_leaf_). In control leaves, g_s_ decreased by 13.6 ± 5.3% over the 90 minutes of measurement, not significantly different from the intercept of fitted non-linear models (16.9 ± 2.3%: Figure 5b). Similarly, the “cuvette effect” decreased transpiration rate of control leaves by 6.1 ± 3.8%, compared with a fitted intercept of 8.1 ± 1.6% (Figure 5c). Variation in transpiration rate due to the cuvette effect, plant-to-plant differences and leaf excision allowed us to explore relationships between leaf temperature and treatment both with (UV+) and without (UV-) UV treatment (Figure 6). Warming due to reduced transpiration, and hence reduced g_s_, was quantified as the slopes of the relationships. Warming independent of stomatal response was quantified as the difference in ΔT_0_ between UV+ and UV-, i.e. the intercept where there is no change in transpiration rate. Analysis of linear regressions fitted to the UV+ and UV- data (Figure 6) showed that the slopes of the UV- and UV+ treatments did not significantly differ (p = 0.19). On that basis, the two treatments shared the same change in ΔT per unit change in transpiration rate, defined by the pooled slope as −0.34°C per mmol m^−2^ s^−1^ (Figure 6). Thus, decreased transpiration resulting from UV exposure would be expected to increase leaf temperature as a function of this slope. However, ΔT_0_ was significantly greater (0.17 ± 0.09°C, p < 0.001) for UV+ than for UV- (Figure 6), demonstrating an additional mechanism of leaf warming in these climate cabinet experiments that augmented the highly significant effect of stomatal closure. We interpret this additional effect as the direct warming effect of the lamps. Subtracting this lamp effect of 0.17°C from the observed increases in ΔT in individual experiments indicates that increases in ΔT due only to UV-induced reductions in transpiration were in the range 0.00 ± 0.14 and 0.71 ± 0.16°C in the climate cabinet experiments.

We used a similar approach to explore whether additional mechanisms of leaf warming occurred in the growth room and field experiments. In the growth room, there were no significant differences in the slopes of the relationships between T_leaf_-T_air_ and transpiration and, in contrast to the climate cabinet experiments, also no significant differences in the intercepts (Figure 4). Similarly, there were no significant differences in either the slope or intercept of the relationships between T_leaf_ and g_s_ in the growth room experiments (Figure 2b) or in the polytunnels (Figure 2a).

The slopes of the relationship between T_leaf_ and g_s_ varied substantially between the three experimental campaigns, as expected from their very different total radiation environments. The greatest leaf temperature increase per unit reduction in g_s_ occurred in the field experiment (0.012°C per mmol m^−2^ s^−1^; Figure 2a), almost seven-fold higher than in the growth room (0.0018°C per mmol m^−2^ s^−1^; Figure 2b), and more than 3-fold higher than in the climate cabinet experiment that delivered the irradiance closest to that in the growth room experiment (0.0036°C per mmol m^−2^ s^−1^; Fig. 2c). This variation is consistent with the much greater total solar radiation measured in the polytunnels in the field experiments (up to approx. 700 W m^−2^ in experiment 2) compared with the climate cabinet (54 W m^−2^) and growth room (485 W per mmol m^−2^ s^−1^ in the cuvette with the LED attachment). Thus, the magnitude of leaf warming in response to the reduction in g_s_ caused by UV radiation varied with total radiation incident on the leaf.

## Discussion

To our knowledge, this is the first report that partial stomatal closure caused by UV radiation significantly increases leaf temperature, up to approximately 1.5°C in our experiments. Multiple experimental conditions and measurement techniques examined whether lamps or filters might alter leaf temperature independently of changes in g_s_, for example by changing air temperature. In the field experiments, significant differences in T_leaf_ between UV+ and UV- tunnels occurred without any significant differences in air temperature. When transient differences in air temperature occurred during leaf temperature measurements, they were small and not significantly correlated with changes in leaf temperature. Furthermore, the slope and intercept of regressions of T_leaf_ against g_s_ in UV+ and UV- treatments (Figure 2a) were not significantly different. Likewise, the slopes and intercepts of regressions of T_leaf_ against g_s_ (Figure 2b) and transpiration rate (Figure 4) for the UV treatments in the growth room experiments (Figure 4) were not significantly different, consistent with the plants being removed from their UV treatment and measured under equal air temperature and radiation load. The significant increase in leaf temperature detected (0.23 ± 0.20°C) in the growth room experiments was therefore independent of any direct heating effect from the UV lamps. In the climate cabinet experiments, we recognized that differences in radiation loading might influence leaf temperature since (i) UV lamps emit radiation >400 nm and (ii) measurements were made under treatment conditions using the cuvette that transmitted UV and PAR from the lamps. However, linear regressions of leaf temperature versus transpiration rate separated the individual warming effects caused by stomatal closure and direct radiative heating, both with and without UV treatment (Figure 6). The highly significant (p < 0.001) difference between leaf warming in the absence of any change in transpiration (ΔT_0_: the Y-intercepts for the two regressions in Figure 6) indicated direct radiative heating of 0.17 ± 0.09°C. Leaf warming due to partial stomatal closure induced by UV radiation was up to 0.71 ± 0.16°C depending on irradiance (0–2.64 Wm^−2^ UV_F&C_).

We also considered whether small changes in air temperature might have influenced g_s_ independent of UV responses by increasing vapour pressure deficit (VPD) which can induce stomatal closure [33–35]. This mechanism could not have occurred in the growth room experiments, since plants were removed from their UV treatment and measured under equal air temperature, VPD and total radiation loading. In the field experiments, UV treatment differences in air temperature had limited impact on VPD (calculated to be approximately 0.2 kPa) and thus were unlikely to induce significant stomatal closure in tomato [34]. Changes in air temperature in the climate cabinet experiments were around an order of magnitude smaller than changes in leaf temperature. Even the largest increase in T_air_ we observed (0.3°C at the end of measurements with our highest UV irradiance) and corresponding reduction in relative humidity (6%, again at the end of measurement) only marginally increased VPD (0.2 kPa under measurement conditions), again much smaller than that required to induce rapid stomatal responses [33–35]. Finally, we explored whether decreased photosynthesis due to UV radiation might have contributed to the observed partial stomatal closure. Across our different experimental regimes and treatments, variable effects on photosynthesis rates (including both small increases and small decreases [21]) were not correlated with changes in leaf temperature. While we cannot exclude the possibility that reduced photosynthesis might be an additional mechanism acting under some conditions [36], direct effects of UV on stomata [11] was the primary mechanism acting in our experiments.

Overall, our data show that leaf warming due to partial stomatal closure induced by exposure to UV radiation is both significant and distinguishable from any warming due to direct radiative effects of lamps or filters. The magnitude of leaf warming observed as a result of UV exposure in our studies is also consistent with the warming predicted by partial stomatal closure caused by other factors. For example, in their investigation of variation between species in stomatal sensitivity to elevated CO_2_ concentration, Idso et al [18] quantified the relationship between partial stomatal closure (expressed as stomatal conductance ratio, in their case conductance in elevated CO_2_/conductance at ambient CO_2_) and leaf warming under field conditions through the following linear regression:

Change in leaf temperature (°C) = 5.40-(6.38 x stomatal conductance ratio)

This relationship predicts that the maximum reduction in conductance observed in our field experiment (156 mmol m^−2^ s^−1^ on day 3, or approximately 0.67 of the control value) should increase leaf temperature by around 1.1°C, broadly similar to the observed increase (Figure 1). Thus, observed increases in leaf temperature are consistent with the expected effect of partial stomatal closure, regardless of any additional radiative heating.

Indeed, well established relationships between g_s_ and leaf temperature [15–20] and the widely reported partial stomatal closure in response to UV radiation (Table 1), raises the question of why this phenomenon has not been reported previously. Possibly it is much harder to show statistically significant leaf warming in controlled environments than in the field, as a result of two inter-related factors. First, the magnitude of leaf warming for a given change in g_s_ varies with total incident radiation [15, 16]. Thus, the magnitude of leaf warming in response to a given change in g_s_ is expected to be much smaller in controlled environments than in the field, as observed (Figure 2). Second, the “signal” of small changes in leaf temperature observed in controlled environments occur against the “noise” of background, short-term variation in air temperature caused by air conditioning units. This low “signal to noise” ratio reduces the likelihood of differences meeting conventional definitions of statistical significance, as evident in our growth room experiments. When leaf warming was corrected for variation in air temperature (T_leaf_-T_air_) the effect of UV radiation was highly significant (p < 0.001: Figure 3b). The same response measured using uncorrected leaf temperature, T_leaf_, as in the field experiments, did not quite meet the p < 0.05 criteria for statistical significance (p = 0.08: Figure 3a). This need to increase the signal:noise ratio led us to use different approaches to measuring leaf warming under our different experimental regimes. Of course, the same issue of low signal to noise ratio may occur in long-term datasets of mean leaf temperature in the field [37]. Possibly other researchers have observed leaf warming due to UV exposure, but not reported it due to lack of formal statistical significance.

Although our data relates to reduced g_s_ in response to UV exposure over hours-days, they agree with abundant evidence from other studies that reduced g_s_ persists in treatments lasting up to several months. Indeed, the reductions in g_s_ observed in our polytunnel experiments (up to 34%) are broadly comparable to reductions of up to 44% reported in the field experiments cited in Table 1. Combined with the well-established role of g_s_ as a partial determinant of leaf temperature [15–20], this suggests that increased leaf temperature will persist beyond the duration of the UV treatments used here. Equally, UV-induced stomatal closure observed in many other species (Table 1) should lead to leaf warming as it does in tomato. Similarly, partial stomatal closure in response to increased CO_2_ concentration also enhanced leaf temperature [18, 38, 39], with between-species variation in the magnitude of stomatal responses determining the magnitude of leaf warming [18]. Furthermore, total solar radiation (as above), VPD and wind speed will all determine the extent to which leaf temperature increases following exposure to UV radiation [15]. For example, although our polytunnels were designed to allow greater air movement than in a fully enclosed structure (Figure S1), less air movement is likely than in an open field, so a given reduction in conductance should increase T_leaf_ to a lesser extent than in a field crop. Nonetheless, our data, and other reports [21, 37], suggest that UV-mediated increases in leaf temperature of up to 2°C might commonly occur in crops growing in the field or well-ventilated protected environments.

The choice of action spectrum used as the BSWF in studies of plant responses to UV remains debatable [5, 40]. The UV_F&C_ irradiances in the field and growth room experiments (maxima approximately 0.48 W m^−2^ and 0.35 W m^−2^ respectively) were within the linear phase of the irradiance response measured in the climate cabinets (approximately 0.50 W m^−2^: Figure 5). Decreased g_s_ in the field and growth room (35% and 21% respectively) experiments agreed with predictions from the modelled irradiance responses (31% and 27% respectively). This broad agreement between field and CE data indicates that using PGIAS as a weighting function successfully accounted for spectral differences between sunlight and artificial UV lamps. This is further corroborated by the observation in the climate cabinets experiments that there were no significant differences between the linear regressions for comparable irradiances (approximately ≤0.3 W m^−2^) delivered using the filtered and unfiltered UV lamps (filled and unfilled symbols in Figure 5: p > 0.2 for all variables) over the range of UV_F&C_ irradiances that could be delivered using either lamps filtered with cellulose diacetate or unfiltered lamps.

Increases in leaf temperature of the magnitude we report here due to UV exposure are sufficient to affect other plant physiological responses. Despite substantial within- and between-species variation, both growth and photosynthesis typically increase with increasing temperature up to an optimum but are inhibited by further increases in temperature. For example, the following relationship between growth temperature and relative growth rate (RGR) in tomato [41].

RGR = -0.2949 + 0.000329*I + (0.04001*T) – (0.000797*(T^2).

(where T is temperature and I is irradiance) can be used to investigate the effects of a 2°C increase in leaf temperature, treating growth temperature as leaf temperature. At 15°C, approximately 10°C below the optimum (25°C), the model indicates an increase of 2°C would increase RGR by 17%. At 35°C, approximately 10°C above the optimum, the same 2°C increase is predicted to decrease RGR by approximately 10%. This prediction of more rapid growth due to leaf warming in the presence of UV radiation agrees with grower reports that crops grew and developed more rapidly early in the growing season when ambient temperatures are cooler [21]. It may also partly account for the very substantial variation in the reported effects of increased UV radiation on photosynthesis and growth under field conditions [42]. On this basis, we predict that negative effects of UV radiation on growth are more likely to be reported under conditions where temperature is high relative to the crop optimum, as the literature suggests [43]. The possibility that exposure to UV radiation exacerbates high temperature stress might also account for the cross-talk between UV and high temperature signaling [44]. Of course, inherent differences in plant UV sensitivity due to differences in plant morphology or UV absorbing pigments [5] will modify the responses of growth or photosynthesis to UV radiation, but we hypothesise that changes in leaf temperature resulting from changes in g_s_ may be an additional or modifying effect.

In conclusion, leaf warming due to partial stomatal closure is a robust response to exposure to UV radiation under a range of experimental conditions. Leaf warming caused by a cellular response to UV radiation (partial stomatal closure), probably mediated via uvr8 [11], appears sufficient to influence “down-stream” whole plant and crop responses. This might have been an additional stress factor for crops and vegetation had the Montreal Protocol not successfully protected crops and vegetation from the effects of both increased UV and climate warming caused by ozone depleting substances, such as chlorofluorocarbons [45]. Nevertheless, the effect of UV on g_s_ and leaf temperature reported here provides further opportunities to exploit UV manipulation in horticultural production, whether using UV-modifying cladding plastics [7, 8] or UV emitting LEDs [9].

## Methods and materials

*Plant Material.* Tomato (*Solanum lycopersicum* cv. “Money Maker”) plants were propagated in a south-facing glasshouse at the Lancaster Environment Centre (Lancaster University; 54°N, 3°W). Temperature was partially controlled by passive ventilation, thermal blinds and heating. Passive ventilation and shading blinds were deployed gradually as ambient total solar irradiance exceeded 600 W m^−2^, and fully deployed when total irradiance reached 1000 W m^−2^. Thermal blinds were deployed when ambient total irradiance fell below 200 W m^−2^ or ambient temperature below 2°C. The minimum glasshouse temperature set point for heating was 15°C and the maximum 24°C. Supplementary light emitting diode (LED) lamps (Senmatic FL300 Grow, Denmark) were switched on when ambient total solar irradiance reduced below 450 W m^−2^ and switched off above 500 W m^−2^, or if glasshouse temperature exceeded 30°C, with a 16-h photoperiod. Seeds were sown in modular tray inserts (15-cell, 7.5 x 7.5 cm cells) containing a peat-based substrate (Levington Advance M3, ICL Everris Ltd, Ipswich). When seedlings reached the 3-leaf stage (~3 weeks old) they were transplanted individually into 2 L (150 mm diameter) pots containing the same substrate. Prior to transfer to their respective experimental environments, plants were selected for uniformity.

### Radiation measurements.

UV and photosynthetically active radiation (PAR: 400-700 nm) were quantified with a double monochromator scanning spectroradiometer (SR9910-V7, Macam Photometrics, Livingston, UK) with cosine head attachment. Following the standard approach used in UV research to account for differences between the spectral balance of UV lamps and sunlight, we express UV treatments as “plant-weighted UV” by using a biological spectral weighting function (BSWF) [40]. BSWFs use action spectra, experimentally derived measures of the effectiveness of specific UV wavelengths in inducing plant responses (E) to “weight” the physical measurement of spectral irradiance at each wavelength. For a given wavelength (λ), the biologically weighted spectral irradiance is the product of effectiveness (Eλ) and spectral irradiance (Iλ). The total biologically weighted irradiance integrated across a waveband, as used here, is then the sum of the individual weighted spectral irradiances. We used the plant growth inhibition action spectrum (PGIAS [46]) as the biological spectral weighting function. Like other plant action spectra, such as the widely used generalised plant action spectrum (GPAS) [47, 48], PGIAS attributes greatest effectiveness to shorter UVB wavelengths. However, in marked contrast to GPAS, PGIAS also attributes substantial effectiveness to UVA wavelengths. Therefore, we refer to PGIAS-weighted UV radiation (= UV_F&C_) rather than UVB radiation. We also describe UV treatments weighted using GPAS (= UV_CALD_) to facilitate comparison with previous studies using that action spectrum.

*UV Treatments and experimental conditions:* (i) Field experiments. Four repeat field experiments were conducted between 17 June and 25 July 2018 using small polytunnels (3.0 m x 1.5 m x 2.25 m tall), also located at the Lancaster Environment Centre. To maximize air circulation, the polytunnels were unclad below the mesh bench where plants were placed, which was 0.75 m above the ground (Figure S1) [49]. Two replicate tunnels were covered with a commercial cladding plastic (Lightworks Sun Master: Arid Agritec Ltd. referred to as “UV-”) that is opaque to wavelengths below approx. 380 nm (Figure S2a). The other two replicate tunnels were clad with a commercial plastic that transmitted at least 40% in the UVB and approx. 75% total of solar UV radiation (Lightworks Sun Smart: Arid Agritec Ltd. referred to here as “UV+”). Claddings were re-randomised between tunnels between the second and third experiments. Spectroradiometric measurements made on a clear sunny day during this experimental period (Figure S2a) showed the UV- cladding transmitted only 3% of UV_F&C_ (0% UV_CALD_) while UV+ transmitted 76% UV_F&C_ (63% UV_CALD_). UV_F&C_ was reduced by approximately 98% in UV- compared with UV+ (Table S6a). Polytunnel air temperature was logged continuously with a TinyTag Ultra 2 data logger (TGU-4017: TinyTag, Gemini Data Loggers, Chichester, UK) hung centrally in each polytunnel 0.3 m above the plants. Ten replicate tomato plants per tunnel were transferred directly from the glasshouse (as above) to the polytunnels at the five-leaf stage (~5 weeks old), and were shielded with UV opaque plastic film during transfer to avoid transient exposure to solar radiation.

*(ii) Controlled Environment (CE) Growth Room experiments.* Three repeat experiments were conducted in a specially designed walk-in growth room. A PAR irradiance of 450 μmol m^−2^ s^−1^ was provided by LEDs (B100 and B150, Valoya, Helsinki, Finland) for a 16-h photoperiod. Unlike other PAR sources, such as the metal halide discharge lamps we have used before [50] and the fluorescent tubes used in the irradiance response studies (see below), these LEDs produce no UVA radiation. Thus, all UV radiation (UVA and UVB: Figure S2b) was provided by specific UV sources that were switched on 1 h after the PAR lamps and switched off 1 h before, giving a “UV day” of 14 h. UVA was provided by Q-Lab UVA-340 fluorescent tubes and UVB by Q-Lab UVB-313 EL fluorescent tubes (both from Q-Panel Laboratory Products, Bolton, UK). The UVB tubes were filtered with 0.13-mm-thick cellulose diacetate (CA: Clarifoil, Courtaulds Ltd, Derby, UK) to remove wavelengths less than approximately 290 nm.

There were two UV radiation treatments: UV+ and UV-. For the UV+ treatment, a layer of the same UV transparent cladding plastic as used in the field (Lightworks Sun Smart) was placed between the experimental plants and cellulose diacetate-wrapped UV sources (as above). This provided 0.35 W m^−2^ s^−1^ UV_F&C_, a daily dose of 17.8 kJ m^−2^ d^−1^ at plant height (0.26 W m^−2^ s^−1^ and 13.0 kJ m^−2^ d^−1^ UV_CALD_ respectively) (Table S6b). The UV_F&C_ irradiance was 26% lower than the maximum irradiance measured in the UV+ tunnel under clear-sky conditions in the field experiments. However, as this irradiance was delivered constantly throughout the 14 h UV day in the growth room, the UV_F&C_ dose was 39% higher than the daily dose measured in the UV+ treatment in the field. For the UV- treatment, a layer of the same UV opaque cladding plastic as used in the field (Lightworks Sun Master) and a layer of UVB-opaque clear polyester (Clear 130, Lee Filters, Andover, UK) were placed between the experimental plants and UV sources (as above). The UV- treatment provided less than 1% of the UV_F&C_ dose provided by the UV+ treatment (Table S6b, Figure S2b). Plant positions were rotated daily to eliminate any effect of small variations in radiation caused by position, each plant spending 24 hours in each of 6 positions within the two UV treatments. In all experiments, growth room temperature was held at 25 ± 1**°**C during the day and 17 ± 1**°**C at night while relative humidity was 54 ± 18% in the day and 52 ± 11% at night. Plants were allowed to acclimate to the growth room environment for 1 week prior to experimentation.

*(iii) Climate cabinet irradiance response experiments*. These experiments were conducted using Microclima 1750 indoor controlled environment cabinets (Snijder Scientific, Tilburg, Holland). The cabinets provided approximately 300 μmol m^−2^ s^−1^ PAR for a 16-h photoperiod (provided by a combination of Sylvania: T5 FHO/54 W/840/1149 mm, T5 FHO/24 W/840/549 mm and Brite GrowT8/58 W/1200 mm fluorescent tubes). UVA radiation (<400 nm) produced by these PAR fluorescent tubes was blocked with UV-O plastic (Lightworks Sun Master), as used in the field and growth room, so that control (UV-) conditions were comparable to our other experiments. To deliver a range of different UV spectra (Figure S2c-f) and UV_F&C_ irradiances, we provided UVB and/or UVA radiation from several different compact fluorescent lamps or fluorescent tubes, with or without filtering by cellulose diacetate (see Table S6c for full details). UV_F&C_ irradiances ranging between 0.008–2.64 W m^−2^ (0–2.55 W m^−2^ UV_CALD_) at the leaf surface (Table S6c) were delivered using these different sources, by raising or lowering the UV radiation source relative to the experimental leaf and by varying the intensity of the UV source. The use of unfiltered lamps was required in our experimental systems to deliver the higher UV_F&C_ irradiances, which were broadly based on the global range modelled using the Atmospheric Chemistry Observations and Modelling Quick TUV model (http://cprm.acom.ucar.edu/Models/TUV/Interactive_TUV/). All UV treatments maintained a constant distance between the plant and the PAR sources. To determine the effects of complete stomatal closure on leaf temperature (thereby providing context for the UV treatments), some leaves were excised to determine the maximum possible leaf warming in this environment.

Air temperature in the climate cabinet was held at 25 ± 1.5**°**C and relative humidity at 60 ± 10%. Plants were transferred from the glasshouse to climate cabinets at the 4-leaf stage (~5 weeks old depending on the season) to acclimate for 1 week before treatments commenced.

*Stomatal conductance, transpiration rate and leaf temperature.* The three different experimental campaigns used different measurement approaches, for both practical reasons and to assess whether the hypothesized effect of UV on leaf temperature was robust in relation to measurement method. Practical reasons included equipment availability at specific times but also the need to make consistent measurements of leaf temperature against the cyclic fluctuations in air temperature that are inherent to the air conditioning systems that provide temperature control in controlled environments.

*(i) Field experiments*. Stomatal conductance (g_s_) was measured using an AP4 leaf porometer (Delta T Devices Ltd, Cambridge, UK). Leaf temperature (T_leaf_) was measured using an infrared thermometer (WZ-39755-10 Deluxe, Cole-Parmer Instrument Company Ltd., St. Neots, UK). All measurements were made on a leaflet from the most recent fully developed leaf pair on the 5th internode (numbering from the base of the plant). Measurements were centered around solar noon, from 11:00 until 15:00, alternating daily the first treatment from which data was collected to minimise diurnal effects confounding UV impacts on the measured variables.

*(ii) Growth room and climate cabinet experiments*. Transpiration rate and g_s_ were measured using a LI-6400 XT (LI-COR Inc., Lincoln, NE, USA). In the growth room, measurements were made using the standard 2x3 cm cuvette with the light emitting diode (LED) attachment (6400-02B LED light source) that provided saturating PAR (1600 μmol m^−2^ s^−1^) but no UV radiation during measurements. Cuvette block temperature was set at 25**°**C, relative humidity at 45–55%, CO_2_ concentration at 400 ppm and flow rate: 500 μmol s^−1^. A leaflet from the most recent fully developed leaf pair on the 3^rd^ internode (numbering from the base of the plant) was used for the growth room experiments. All leaf gas exchange measurements were centered around the middle of the photoperiod to minimise the effects of diurnal variation in g_s_. After enclosing a leaf inside the cuvette, the internal environment and gas exchange parameters were allowed to stabilise (1–2 minutes) before recording data.

In the climate cabinet experiments, following acclimation to the cabinet environment, plants were transferred to a second, adjacent climate cabinet where individual plants were exposed to UV treatments on a replicate-by-replicate basis. A leaflet from the youngest fully developed leaf pair on the 5^th^ internode (numbering from the base of the plant) was used for gas exchange measurements. The LI-COR 6400 XT was used with the standard 2x3 cm cuvette and the clear (“propafilm”) cuvette top attachment that transmitted both PAR and UV radiation from the cabinet, allowing approximately 300 μmol m^−2^ s^−1^ PAR. The cuvette block temperature was 25**°**C, relative humidity ranged 45–55%, CO_2_ 400 ppm and flow rate 500 μmol s^−1^. For each replicate, after the leaf was enclosed in the cuvette, data was logged every 10 seconds and the internal environment and gas exchange parameters were allowed to stabilise for the initial 15 minutes. After stabilising, either the same conditions were maintained for 90 minutes (controls), or UV radiation applied for 90 minutes, or the leaf excised. Gas exchange data from the climate cabinet experiments was recorded as the mean percentage change over the 90-minute treatment period.

In the growth room and climate cabinet experiments, the built-in thermocouple of the LI-6400 XT cuvette measured leaf temperature. Even using the cuvette, short-term (typically 10–15 minutes) cycles in growth room or climate cabinet air temperature of up to 1.2**°**C partially obscured the changes in leaf temperature due to variation in g_s_. In the growth room experiments, as well as measuring T_leaf_, leaf temperature responses to UV treatments were expressed as the difference between T_leaf_ and the concurrent air temperature measurement (T_air_), referred to here as T_leaf_-T_air_. In the climate cabinet irradiance response experiments, the need to track leaf temperature changes in response to short term UV treatments prompted a different approach:

$$ \varDelta T={\left({T}_{leaf}-{T}_{air}\right)}_{FINAL}-{\left({T}_{leaf}-{T}_{air}\right)}_{START} $$

where (T_leaf_-T_air_)_FINAL_ is the difference between leaf and air temperature at the end of UV treatments (the short term analogue of T_leaf_-T_air_ in the growth room experiments) and (T_leaf_-T_air_)_START_ is the difference at the start of treatment. As for gas exchange (see above), the leaf was enclosed in the cuvette and allowed to stabilise for 15 minutes. T_leaf_ and T_air_ were logged continuously during the 90 minute treatment period. Example time courses of all three treatments (control, excised leaves, UV radiation - Figure S3) demonstrate the typical leaf temperature response and how ΔT was derived from T_leaf_ and T_air_.

*Data analysis*. Data from field and controlled environment campaigns were statistically analysed for differences between the UV treatments (UV+ / UV-) using a repeated measures ANOVA in SPSS version 18 (SPSS Inc. Chicago, USA), with UV treatment and experiment as the main factors and day as the repeated measure. Tunnel was also included as a main factor in the analysis of data from the field campaign.

The relationships between UV_F&C_ irradiance and changes in leaf temperature, g_s_ and transpiration rate in the climate cabinet campaign were analysed using non-linear regression in GraphPad Prism version 7.0d for Mac OS X (GraphPad Software, La Jolla California USA, www.graphpad.com). We initially tested whether relationships were best fitted using a linear model or a non-linear model. The alternative models were as follows:

(1) \begin{equation*} Linear:{R}_I={R}_0+\left(K\ x\ I\right) \end{equation*}

(2) \begin{equation*} Non- linear:{R}_I={R}_0+(\left({R}_{max}-{R}_0\right)\ x\ (1-\mathit{\exp}(-K\ x\ I))) \end{equation*}

In both models, R_I_ is the response at irradiance I, the intercept (R_0_) is the response at zero UV_F&C_ and K is an irradiance response constant (a measure of the unit response per unit UV_F&C_). In the non-linear model the response reaches a plateau at R_max_. The non-linear model provided a significantly better fit than the linear model (p = 0.018 and p = 0.019 for leaf temperature and transpiration rate respectively). The non-linear model also gave the better fit for g_s_ but this was not quite statistically different from the linear model (p = 0.06). However, since the non-linear model gave a significantly better fit for transpiration rate, non-linear models are presented for all three parameters.

Relationships between leaf temperature and g_s_ or transpiration rate under different experimental conditions were further explored using linear regressions. In particular, this approach quantified any effects of UV treatments on leaf temperature that were independent of stomatal responses (e.g. an effect of any heat output from the UV source). The relationship between changes in transpiration rate and leaf temperature were presented separately for treatments with and without UV radiation. Warming independent of stomatal response was quantified as the difference in the Y intercept (i.e. with no change in transpiration rate). The magnitude of changes in the Y intercept and its statistical significance was determined using GraphPad Prism version 7.0d.

## Acknowledgments

This research was supported by a UK Natural Environment Research Council studentship (Ref 1653788) to Tom Williams. We thank Robert Kempster for help re-cladding the polytunnels, Maureen Harrison for technical assistance, Dr. Samuel Taylor with the leaf gas exchange measurements, and Dr. Jason Moore for many helpful discussions.

## Author contributions

Concept and design (NDP, WYS, ICD), experiment design (TBW, NDP), data acquisition (TBW), data analysis (TBW, NDP), data interpretation (TBW, NDP, ICD), manuscript drafting (TBW, NDP), manuscript revision (TBW, NDP, ICD), manuscript approval (TBW, NDP, ICD, WYS).

## Data availability

The raw data on which the manuscript is based has been provided as supplementary files and referenced within the text where appropriate.

## Conflict of interest statement

The project was initiated by repeated anecdotal reports from commercial growers in Turkey that crops grew more rapidly and matured earlier under ultraviolet transparent polytunnel claddings than under conventional claddings. Those reports from growers were to one of our authors, Dr. Wagdy Sobeih, who is Managing Director of Arid Agritec, a company that supplies cladding materials to growers. As such, we feel it is appropriate to disclose this as a potential conflict of interest. We declare that there is no conflict of interest for the other authors of this manuscript.

## Supplementary data

Supplementary data is available at *Horticulture Research Journal* online.

## Supplementary Material

Web_Material_uhab066
