# Supplementary material for: Ultraviolet radiation causes leaf warming due to partial stomatal closure
Source: Hortic Res. 2022 Jan 19;9:uhab066. doi: 10.1093/hr/uhab066 (PMC8944299; doi:10.1093/hr/uhab066)
Supplement: Web_Material_uhab066 [file web_material_uhab066.docx]

**SUPPLEMENTARY INFORMATION**

**Table S1. Polytunnel multi-day data.** Polytunnel raw data supplied as a separate Excel file.

**Table S2a. Combined field experiments statistics summary.** p values from the repeated measures analysis of variance of leaf temperature (*T_leaf_*) and stomatal conductance (*g_s_*) data from the four field experiments combined together as one data set.

| **ANOVA Factors** | ***T_leaf_*** | ***g_s_*** |
| --- | --- | --- |
| Treatment | **<0.001** | **0.003** |
| Experiment | **<0.001** | **<0.001** |
| Polytunnel | **0.045** | 0.833 |
| Day | **<0.001** | **<0.001** |
| Treatment x Experiment | 0.131 | **0.001** |
| Treatment x Polytunnel | 0.306 | 0.435 |
| Treatment x Day | 0.436 | 0.188 |
| Experiment x Polytunnel | 0.271 | 0.211 |
| Experiment x Day | **<0.001** | **<0.001** |
| Polytunnel x Day | 0.141 | **0.041** |
| Treatment x Experiment x Polytunnel | **0.001** | 0.544 |
| Treatment x Experiment x Day | **0.030** | **0.010** |
| Experiment x Polytunnel x Day | **<0.001** | 0.337 |
| Treatment x Polytunnel x Day | **0.002** | 0.128 |
| Treatment x Experiment x Polytunnel x Day | **<0.001** | 0.164 |

**SUPPLEMENTARY INFORMATION**

**Table S2b**. **Individually analysed** **field experiment statistics summary**. p values from repeated measures analysis of variance of leaf temperature (T_leaf_) and stomatal conductance (g_s_) data from the field experiment completed when conditions were most consistently cloud-free (25 June – 3 July 2018).

| **ANOVA Factors** | ***T_leaf_*** | ***g_s_*** |
| --- | --- | --- |
| Treatment | **0.001** | **0.006** |
| Polytunnel | 0.183 | 0.175 |
| Day | 0.133 | **<0.001** |
| Treatment x Polytunnel | **0.017** | 0.421 |
| Treatment x Day | 0.993 | 0.716 |
| Polytunnel x Day | 0.338 | 0.390 |
| Treatment x Polytunnel x Day | 0.710 | 0.313 |

**SUPPLEMENTARY INFORMATION**

**Table S3. Controlled environment (CE) growth room multi-day data.** CE growth room raw data supplied as a separate Excel file.

**Table S4. Controlled environment growth room statistics summary.**  p values from repeated measures analysis of variance for each leaf physiological parameter measured in the three repeat experiments in the controlled environment growth room, combined into a single dataset.

| ANOVA Factors | *T_leaf_* | *T_leaf_ - T_air_* | *E* | *g_s_* |
| --- | --- | --- | --- | --- |
| Treatment | **0.081** | **0.007** | **0.002** | **0.008** |
| Experiment | **<0.001** | **<0.001** | **<0.001** | **<0.001** |
| Day | **0.006** | **0.003** | **<0.001** | **<0.001** |
| Treatment x Experiment | 0.618 | 0.489 | 0.684 | 0.804 |
| Treatment x Day | 0.431 | 0.140 | **<0.001** | **<0.001** |
| Experiment x Day | **<0.001** | **<0.001** | **<0.001** | **<0.001** |
| Day x Experiment x Treatment | 0.560 | 0.428 | 0.083 | 0.159 |

**Table S5. Climate cabinet 90 min irradiance response data.** Climate cabinet raw data supplied as a separate Excel file.

**SUPPLEMENTARY INFORMATION**

**Table S6. The experimental ultraviolet irradiances and doses.** Irradiances used in (a) polytunnel, (b) controlled environment room and (c) the climate cabinet irradiance response experiments. Data are expressed as plant weighted UV radiation using the plant growth inhibition action spectrum (UV_F&C_ ^46^). Daily integrated UV_F&C_ doses are also given for the field and growth experiments, but not for the CE cabinets with 90 minute treatments. Data for the field experiments are maxima measured under clear sky conditions.

a) Polytunnel experiments in the field

| Treatment | UV_F&C_ Weighted Irradiance  (mW m^-2^) | UV_F&C_ Weighted Dose  (kJ m^-2^) |
| --- | --- | --- |
| UV+ | 476 | 12.8 |
| UV- | 14 | 0.4 |

b) Controlled environment growth room experiments

| Treatment | UV_F&C_ Weighted Irradiance  (mW m^-2^) | UV_F&C_ Weighted Dose  (kJ m^-2^) |
| --- | --- | --- |
| UV+ | 354 | 17.8 |
| UV- | 2 | 0.1 |

c) Climate cabinet irradiance response experiments

| Lamp type (and filter for UVB sources) | Weighted Irradiance  (mW m^-2^) |
| --- | --- |
| Control (no lamp) | 0 |
| UVA compact fluorescent tube^1^ | 8 |
| UVA compact fluorescent tube^1^ | 37 |
| UVB fluorescent tube^3^, filtered with cellulose diacetate | 97 |
| UVB compact fluorescent tube^2^ | 102 |
| UVA fluorescent tube^4^ | 111 |
| UVB fluorescent tube^3^, not filtered with cellulose diacetate | 155 |
| UVB fluorescent tube^3^, filtered with cellulose diacetate | 208 |
| UVB fluorescent tube^3^, filtered with cellulose diacetate | 251 |
| UVB compact fluorescent tube^2^ | 259 |
| UVB fluorescent tube^3^, filtered with cellulose diacetate | 297 |
| UVB fluorescent tube^3^, not filtered with cellulose diacetate | 300 |
| UVB fluorescent tube^3^, not filtered with cellulose diacetate | 707 |
| UVB fluorescent tube^3^, not filtered with cellulose diacetate | 1,120 |
| UVB fluorescent tube^3^, not filtered with cellulose diacetate | 1,798 |
| UVB fluorescent tube^3^, not filtered with cellulose diacetate | 2,640 |

^1^. Helix 25W Black Light Blue UV, Prolite, Ritelite Systems Ltd, Stamford, UK

^2^. ZooMed ReptiSun 10.0 UV-B Desert, ZooMed Laboratories Inc., San Luis Obispo, USA

^3^. Q-Lab UVA-340, Q-Panel Laboratory Products, Bolton, UK

^4^. Q-Lab UVB-313 EL Q-Panel Laboratory Products, Bolton, UK

See Figure S2 for details of the lamp spectra

**SUPPLEMENTARY INFORMATION**

**Figure S1**: Polytunnel structure. A small (3.0 x 1.5 m x 2.25 m tall) metal frame (A), on supporting plates (B), with steel mesh bench for plants positioned 0.75m above ground (C), and space at each end for working (D). Polytunnel cladding was fitted down to the bench level to facilitate airflow inside^49^.


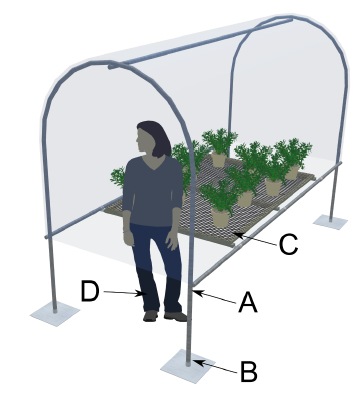


**SUPPLEMENTARY INFORMATION**

**Figure S2**: (a) Spectral transmission (260-700 nm) of the UV transparent (UV+; Lightworks Sun Master) and UV opaque (UV-; Lightworks Sun Smart: Arid Agritec, Lancaster UK) plastic materials used to clad the polytunnels in the field experiments. (b) Spectral irradiance of the UV+ (solid line) and UV- (dotted line) treatments provided by a combination of Q-Lab UVA340 and cellulose diacetate filtered Q-lab UVB313 fluorescent tubes in the growth room experiments. (c-f) Spectral irradiance of the ultraviolet (UV) sources used in the growth room and CE cabinet experiments. (c) Q-lab UV-A 340 fluorescent tube (growth room and CE cabinet), (d) Q-lab UV-B 313 fluorescent tube, unfiltered (solid line: CE cabinet only) and filtered with cellulose diacetate (dotted line CE cabinet and growth room), (e) UV-A compact fluorescent lamp (CE cabinet only), and (f) UV-B compact fluorescent lamp (CE cabinet only).

**SUPPLEMENTARY INFORMATION**

**Figure S3**: Example time courses of T_leaf_, T_air_ and the resulting *ΔT = (T_leaf_-T_air_)_FINAL_ - (T_leaf_-T_air_)_START_* (°C) for (a) control, (b) leaf excision and (c) UV treatments. At zero minutes the leaf was enclosed in the LI-COR 6400 XT cuvette and data logging started. The conditions inside the cuvette were allowed to stabilise for 15 minutes without further treatment. After 15 minutes (vertical dashed line), the treatment was maintained (a), the leaf was excised (b) or UV was applied (c) and continued for 90 minutes. In control leaves (a) both and T_leaf_ and T_air_ remained relatively stable resulting in a stable T_leaf_-T_air_. In leaves excised from the plant (b) T_air_ remained stable while T_leaf_ (and so T_leaf_-T_air_ ) progressively increased which gradually plateaued after 45 minutes. This demonstrates that the technique detects the brief and rapid T_leaf_ increase resulting from transpiration decline associated with leaf excision. When UV was applied (c), T_air_ fluctuated slightly (±0.2°C) which influenced the measured increase in T_leaf_ and T_leaf_-T_air_. This illustrates why *ΔT = (T_leaf_-T_air_)_FINAL_ - (T_leaf_-T_air_)_START_* (°C) must be determined to avoid any effect of air temperature on leaf temperature.
